# Management of Littre Hernia—Case Report and Systematic Review of Case Reports

**DOI:** 10.3390/jcm12113743

**Published:** 2023-05-29

**Authors:** Marian Răcăreanu, Silviu Daniel Preda, Agnesa Preda, Victor Dan Eugen Strâmbu, Petru Adrian Radu, Tudor Constantin Bratiloveanu, Ștefan Pătrașcu, Daniela Marinescu, Konstantinos Sapalidis, Valeriu Șurlin

**Affiliations:** 1 Faculty of Medicine, Department of Surgical Specialities, University of Medicine and Pharmacy of Craiova, 200349 Craiova, Romania; dr.marianracareanu@yahoo.com (M.R.); c_brati@yahoo.com (T.C.B.); stef.patrascu@gmail.com (Ș.P.); dtmarinescu@yahoo.com (D.M.); sapalidiskonstantinos@gmail.com (K.S.); vsurlin@gmail.com (V.Ș.); 2 Craiova Emergency Clinical Hospital, 210218 Craiova, Romania; agnesapcela@gmail.com; 3 Clinic of Surgery of “Dr. Carol Davila” Nephrology Clinical Hospital, University of Medicine and Pharmacy “Carol Davila” Bucharest, 050474 Bucharest, Romania; drstrambu@yahoo.com (V.D.E.S.); drradupetru@yahoo.com (P.A.R.); 4 Third Surgery Clinic, Aristotle University of Thessaloniki, 541 24 Thessaloniki, Greece

**Keywords:** Littre hernia, meckel, diverticulum, case, report, systematic review, outcomes, bowel

## Abstract

Littre hernia is a rare type of hernia in which a Meckel diverticulum is found in the hernia sac. Given the rare nature of this disease, little data on demographics and surgical management exists. In this article, we provide a case report of a strangulated inguinal Littre hernia and perform a systematic review of the literature. The PubMed database was searched on 5 March 2022, and all cases of Littre hernia in adults that had English abstracts or full-text were analyzed. Our primary objective was to evaluate the surgical management and outcomes of this particular type of hernia, and our secondary objectives were to assess demographic characteristics, presentation particularities, and recurrence rates. We identified 89 articles with 98 cases, including our own. Results show a high prevalence of complications described intraoperatively, with strangulation being present in up to 38.46% of patients. The laparoscopic approach was utilized in patients with femoral, inguinal, and umbilical hernias. The most commonly performed type of resection was MD resection, followed by bowel resection, while a minority of cases (5.48%) remained unresected. Mesh repair was more frequently performed in patients with MD resection. A mortality rate of 8.7% in patients who underwent bowel resection was found. A relatively high number of reports of ectopic tissue (21.21%), ulceration (12.12%), and tumors (9.09%) were found. The average follow-up was 19.5 ± 10.29 months, with no hernia recurrence. In conclusion, most cases are admitted in an emergency setting, and intestinal obstruction is frequently associated. A minimally invasive approach can be an option even for complicated hernias. MD resection or bowel resection is usually employed, depending on the extent of ischemic lesions. Patients undergoing bowel resection may be prone to worse outcomes.

## 1. Introduction

Meckel’s diverticulum (MD) is a saccular formation present in the lower part of the intestine, and it is considered one of the most common congenital anomalies of the gastrointestinal tract [1,2]. It results from embryologic closure failure of the omphalomesenteric duct [3]. The protrusion of MD in a hernia sac is called a Littre hernia [2]. The name traces its origins to Alexis Littre, who first described this disease in the 18th century. This type of presentation is rare; only a few cases have been reported in the literature. In this article, we present a case of a strangulated Littre hernia and perform a systematic review of the literature.

The primary objective of this study was to evaluate the surgical management and outcomes of this particular type of hernia, and secondary objectives were to assess demographic characteristics, presentation particularities, and recurrence rates.

## 2. Materials and Methods

### 2.1. Case Report

Ethical Committee approval for the publication of this case report was obtained from the University of Medicine and Pharmacy of Craiova and from the Craiova Emergency Clinical Hospital (No. 119/17 December 2018). Patient consent for publication was also obtained.

For the reporting of the case report, the CAse REport Guidelines (CARE) were followed, and the CARE checklist was completed [4].

### 2.2. Systematic Review

Given the rare occurrence of this disease, a thorough literature search and analysis were carried out to identify optimal management strategies. As case reports constitute the predominant form of evidence available for this condition, an extensive scoping of the literature was performed to identify as many single patient data reports as possible. The scoping of the literature also revealed the existence of previously performed systematic reviews. While a relatively recent systematic review exists, the focus of the paper was primarily on presentation data, and further information regarding surgical management was deemed necessary. Consequently, this study aims to expand the knowledge base on Littre hernia by providing additional insights into its surgical management.

This article adhered to the PRISMA guidelines for reporting systematic reviews [5], and the PRISMA checklist was completed for the manuscript and abstract.

**Figure 1:**
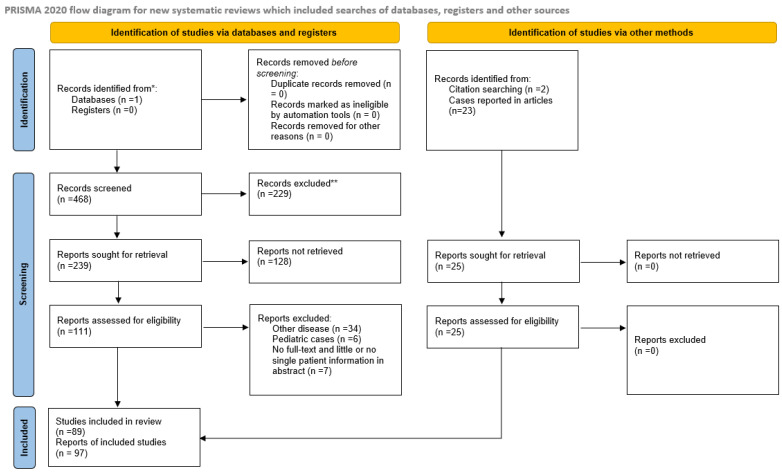
PRISMA flowchart. *, ** Flow of information through the different stages of the systematic review.

A systematic review was conducted on the PubMed database (ncbi.nlm.nih.gov) utilizing a search syntax composed of the following terms: “Meckel”, “diverticulum”, “Littre”, “hernia”, and other related synonyms (Appendix A). The PubMed database was searched on 5 Mar 2022 and the search yielded 468 results, which were imported into Rayyan [6]. Supplementary, all articles were scoped for additional records. During the retrieval of articles, we identified two additional articles that were included in the analysis (Figure 1). Two independent, blinded reviewers, DP and AP, performed the screening of the articles. A third unblinded reviewer (MR) adjudicated discrepancies.

Eligibility criteria for inclusion encompassed articles written in English for adult populations over 18 years of age and the presence of a Meckel diverticulum herniation. Exclusion criteria encompassed articles written in languages other than English, articles reporting on cadavers, articles reporting on children, and articles where neither full-text nor abstract were found. If an abstract was found and provided the minimum information, the case was kept for transparency. After screening, the remaining articles were full-text assessed, and the data was compiled into a Microsoft Excel spreadsheet (Appendix B, Spreadsheet). Articles describing internal hernias were also excluded.

### 2.3. Data Interpretation

Interpretation of data may be subject to bias, prompting the need to define certain aspects and maintain transparency over the interpretation process. In regards to the admission settings of the patients, when no indication was provided for elective or emergency admission, the data was recorded as ND (no data). For clinical complications, our assessment focused on whether the hernia was strangulated, incarcerated, or if an enterocutaneous fistula was present. We have identified inconsistencies in the definitions of the aforementioned notions. We noted as “incarcerated”, those hernias in which the authors specified this term, but in cases in which necrotic bowel or MD was discovered, we noted “strangulation”. With regard to bowel obstruction, we either searched for the term in the article or interpreted the diagnosis in cases where imagistical findings and symptoms supported this diagnosis. The viability of the MD was based on pathology reports and images in cases where the authors did not specify, while lack of information was indicated as ND. For all umbilical hernias resolved with open approach laparotomy, yes was marked. In cases of resections, cuneiform resections of the MD and diverticulectomy were marked as resection of the MD. We could not treat them separately due to inconsistency in reporting, though an analysis of the type of resection used with regards to the width of the diverticulum could have been beneficial. Repair techniques were initially retrieved, but as no recurrences were reported, a simpler classification of anatomic/mesh repair was adopted. In cases where two-stage repairs were used, only the first procedure was noted.

The above data Interpretation was established prior to the full-text assessment of articles.

There seems to be misinterpretation of notions in the literature, which is why we decided to use the following definitions: Littre hernia—hernia that contains a Meckel diverticulum; incarceration of hernia—hernia complication that results in the impossibility of reducing the hernia; strangulation—hernia complication caused by interruption of blood supply to the herniated organ, resulting in ischemia, necrosis, or gangrene [7].

## 3. Case Report

### 3.1. Presentation

A 51-year-old Caucasian male presented to the emergency department of the Craiova Emergency Clinical Hospital in Romania with a sudden onset of symptoms 24 h prior to presentation, in the absence of physical effort. The symptoms consisted of diffuse abdominal pain and the appearance of a round, painful bulge in the right inguinal region. Bowel movement and gas passage were reported as being present.

### 3.2. Medical, Family, and Psychosocial History

Previous medical history was unremarkable patient did not have any current diseases, did not take any ambulatory medication, and had never suffered a surgical intervention. Family and psychosocial histories were also deemed unremarkable.

### 3.3. Clinical Examination

Clinical examination of the patient found a pseudo-tumoral bulge in the right inguinal region, with a firm consistency, painful both spontaneously and at taxis, with absent impulsion and expansion at cough, irreducible at gentle taxi maneuvers, and normal overlaying skin. The abdomen was not distended and was mobile with respiratory incursions, although it was painful both spontaneously and at deep palpation in the lower quadrants of the abdomen with no guarding or muscle contracture.

The rectal examination was normal, with normal stool consistency.

### 3.4. Paraclinical Investigations

The blood work-up showed no abnormalities, with no leukocytosis or anemia.

The patient was admitted with a clinical diagnosis of strangulated right inguinal hernia. No additional imaging was deemed necessary.

### 3.5. Surgery

**Figure 2:**
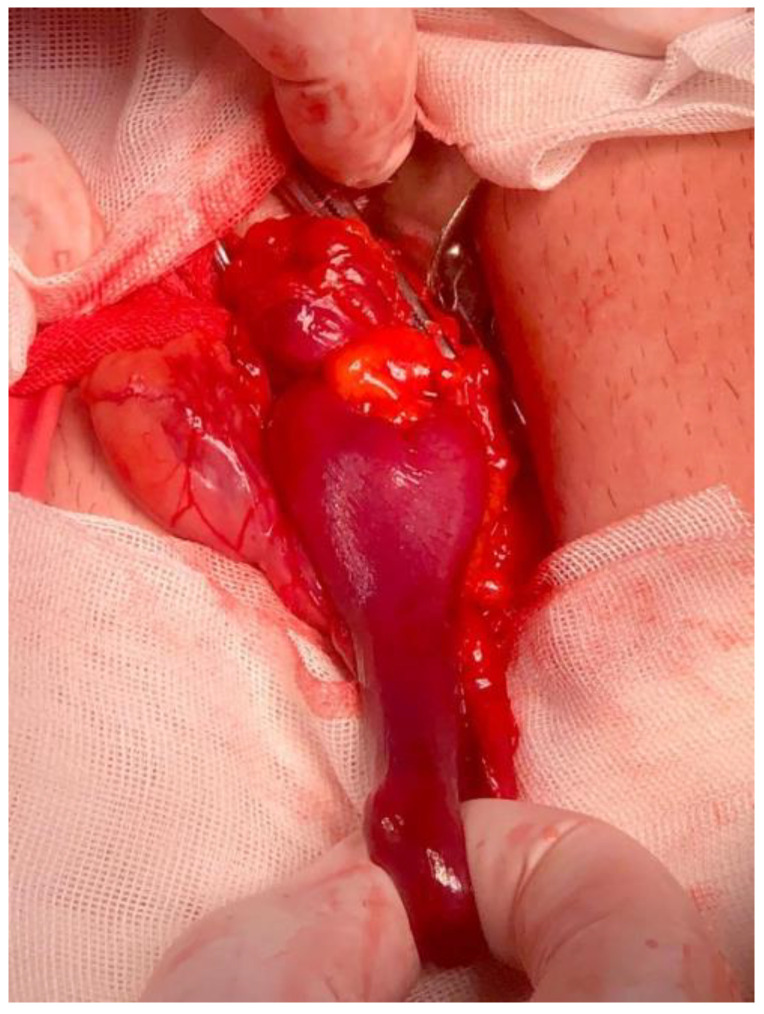
Intraoperative aspect of strangulated Meckel diverticulum.

The patient was transferred to the operating theater and underwent emergency surgery for a strangulated right inguinal hernia. An 8 cm oblique incision in the right inguinal region was made, and after opening the inguinal canal, an external oblique hernia sac of approximately 8/5 cm was found. The sac was isolated from the spermatic cord and opened, with the evacuation of 30 mL of hemorrhagic liquid. After liquid aspiration, a strangulated, non-viable, 6/2 cm Meckel diverticulum was identified, with strangulation markings at the base of the diverticulum (Figure 2). Manual diverticulectomy was practiced, the sac was ligated and reduced, and an anatomical hernia repair (Bassini technique) was carried out.

The postoperative course was uneventful; the patient was discharged on the 5th postoperative day with active mobility, oral intake, and stool passage present. Antibiotics were administered only intraoperatively (one dose of i.v. Cefuroxime 2 g) and were not continued in the postoperative period.

Pathological examination reveals a small intestine mucosa with hypertrophia of lymphoid follicles, edema in the submucosa, and intramural hemorrhagic spots.

Follow-up

The patient was followed-up postoperatively for 2 years in the ambulatory setting (visits at 1, 3, 6, and 12 months) and every 6 months thereafter via telephone.

Currently, the patient is 28 months after surgery with no signs of recurrence or chronic pain.

## 4. Results

The search yielded 89 articles with 98 cases of Littre hernia.

### 4.1. Gender

Gender distribution was found to be slightly higher among females (*n* = 53), with a ratio of 1.2:1. Femoral hernias were more prevalent in female patients (34:14, ratio 2.4:1), while inguinal hernias were more common in male patients (21:6, ratio 3.5:1). Bowel obstruction was more frequent in female patients (18 vs. 8, ratio 2.25:1) but without reaching statistical significance (OR 2.3478, 95% CI: 0.85 to 6.44, *p* = 0.0978). Regarding the postoperative course, death occurred in 11 patients (11.22%), with no statistical significance between female and male mortality (7 female patients and 4 male patients, OR 1.45; 95% CI: 0.99 to 5.28; *p* = 0.57).

### 4.2. Age

The average age of patients was 56.69 ± 16.87 years, with a slightly older female population (57.94 ± 15.33 vs. 55.73 ± 18.31; Mann-Whitney U test *p* = 0.58). The average age of patients with obturator hernias was 73.33 ± 2.36 years, which is consistent with the “little old lady hernia” name given to these hernias. Patients with bowel resection were of slightly older age compared to patients with MD resection or cases in which MD was preserved (61.7 ± 16.4 vs. 56.17 ± 17.76 vs. 55.75 ± 7.08). Slight differences in age can also be observed in patients who underwent mesh repair of the hernia (56.05 ± 14.73 years) compared to patients who underwent anatomic repair (62.06 ± 16.29 years).

### 4.3. Presentation

Data from presentation settings were available for 61 out of 75 cases (92.76%) and show a very large predominance of 90.2% (*n* = 55) of emergency cases, which may explain the high global mortality of 11.22% (*n* = 11), although nine of these patients were from 1700 to 1933.

### 4.4. Location

Femoral hernias (50%, *n* = 49) were the most frequent type, followed by inguinal (27.55%, *n* = 27) and umbilical (11.22%, *n* = 11) hernias. Rare types of Littre hernia were obturator (3.06%, *n* = 3), Spiegel (2.04%, *n* = 2), and transthoracic (2.04%, *n* = 2). The majority of hernias were located on the right side (78.3%, *n*= 54), with the median (15.9%, *n* = 11) and left (5.8%, *n* = 4) sides being less frequent.

Signs of clinical strangulation were observed in half of the cases (*n* = 36/72).

### 4.5. Bowel Obstruction

At presentation, bowel obstruction was present in 35.62% of patients (*n =* 26/73). All obturator hernias presented with bowel obstruction. Inguinal hernias had the highest rate of bowel obstruction (45%, *n* = 9), followed by femoral hernias (30.3%, *n* = 10) and umbilical hernias (*n* = 1, 11.11%). Left-sided hernias had the highest association with bowel obstruction (75%, *n* = 3/4), followed by right sided hernias (34.1%, *n* = 14/41) and median hernias (11.1%, *n* = 1/9).

### 4.6. Imaging

The most commonly used imaging investigation was X-ray (56.14%), although it was often used in conjunction with other imaging investigations such as computed tomography (17.54%) or ultrasound (8.77%) (Table 1).

### 4.7. Surgical Findings

The Meckel diverticulum was found to be viable in 26.53% of cases (*n* = 26/61). The average length of the Meckel diverticulum was 5.43 ± 3.67 cm.

A high prevalence of complications was described intraoperatively, with strangulation being present in up to 38.46% of patients (Table 2). Gangrenous lesions were observed in either the Meckel diverticulum or the strangulated bowel in 25.93% of patients (14/54).

With respect to surgical approach, laparotomy was selected in a considerable proportion (44.78%) of patients, including patients undergoing repair for umbilical and ventral hernias. Further analysis by hernia site reveals a notably elevated utilization of laparotomy in patients with inguinal or femoral hernias (30.43% and 32%) (Table 3).

The laparoscopic approach was utilized in patients with femoral (8%, *n* = 2/25), inguinal (4.35%, *n* = 1/23), and umbilical (20%, *n* = 2/10) hernias. However, it is noteworthy that none of these patients presented with bowel obstruction. On the other hand, laparotomy was more frequently used in patients with bowel obstruction (77.78%) compared to those without bowel obstruction (31.58%) (OR: 6.125; 95% CI: 1.6390 to 22.8891; z statistic 2.695, *p* = 0.007). No significant differences were found between MD viability and laparotomy (OR 2.4; 95% CI: 0.69 to 8.40; z statistic 1.37, *p* = 0.17).

Mesh repair was most prevalent in patients undergoing laparoscopic approaches (80%, *n* = 4/5), and was the least used in patients undergoing laparotomies (23.81%, *n* = 5/30).

### 4.8. Surgical Attitude

The most commonly performed type of resection was MD resection, followed by bowel resection, while a minority of cases (5.48%) remained unresected (Table 4). With regards to the employed technique, manual resection and anastomosis were utilized in the majority of cases (57.14%). Although anatomic repair was the most prevalent, the utilization of mesh for the repair of Littre hernias was also notable, accounting for 32.97% of cases.

In patients undergoing bowel resection, staple and manual resection were used in almost equal proportions (45.45% vs. 54.55%). In patients undergoing diverticulectomy, manual resection is more frequent than staple resection (19 vs. 11 cases). Furthermore, it was observed that the proportion of patients subjected to anastomosis accounted for 81.8% of the patient population (*n* = 18/22) (Table 5).

In terms of repair, mesh repair was more frequently performed in patients with MD resection in comparison to patients who received bowel resection (12 vs. 2 cases) (OR 4.7727, 95% CI 0.9441 to 24.1284; z statistic 1.89; *p* = 0.0587) (Table 6).

The distribution of postoperative outcomes over resection type reveals a mortality of 8.7% in patients who underwent bowel resection, while no mortality was recorded in patients undergoing MD resection (Table 6). Similarly, postoperative discharge duration is higher in patients undergoing bowel resection compared to patients undergoing MD resection (13.6 ± 26.24 vs. 8.35 ± 7.06) (Table 7). Mesh repair was associated with a shorter postoperative stay compared to anatomic repair (3.73 ± 1.73 vs. 9.79 ± 7.04) (Table 7).

Bowel obstruction was associated with a mortality rate of 28%, compared to 0% in patients without bowel obstruction (Table 8).

From available pathology reports, we analyzed the occurrence of ulceration, the presence of ectopic tissue, and the presence of tumors. A relatively high number of reports of ectopic tissue (21.21%), ulceration (12.12%), and tumors (9.09%) were found (Table 9).

### 4.9. Postoperative Course

The postoperative course was uneventful for the majority of cases (69.23%), but mortality is high, occurring in 12.09% of cases. Rates for wound infection (3.3%), wound dehiscence (1.1%), and seroma (1.1%) are low but should be taken into consideration for the placement of mesh. The average postoperative stay was 9.71 ± 15.59 days. No cases of postoperative bleeding or leakage were recorded.

Follow-up is scarce. Only 8 cases (8/98, 8.16%) are followed up for more than 12 months. From available data, the average follow-up was 19.5 ± 10.29 months, with no hernia recurrence.

## 5. Discussion

The present article offers a case report of a rare Littre hernia with a strangulated MD and its surgical management, with an adequate follow-up of 24 months.

The gender distribution in our review was very different from the literature data. Our search identified a male-to-female ratio that was almost equal, while other studies report a female predominance [8]. Moreover, we did not discover statistical differences in terms of bowel obstruction in male vs. female patients or in mortality. This may indicate that gender may not be a significant factor in determining prognosis.

The presentation of the majority of cases included was in the emergency department due to frequent presentations with complications such as enteroaeric fistula or intestinal obstruction.

In regards to hernia location, previous studies report the majority of Littre hernia occurring in the inguinal region, followed by the femoral and umbilical regions, whereas our review portrays a majority of cases in the femoral region, followed by the inguinal and umbilical regions [2,9,10,11].

The clinical diagnosis of a Littre hernia is next to impossible. In most cases, authors suspect a Richter hernia. Nevertheless, the diagnosis of a strangulated or incarcerated hernia is clinical, and in most cases, so is the indication for surgery. The use of imaging is helpful as a decision-to-treatment aid, such as for the diagnosis of intestinal obstruction or hernia complications. WSES guidelines for emergency repair of complicated abdominal wall hernias recommend emergency surgery when intestinal strangulation is suspected [12]. The international guidelines for groin hernia management state that clinical examination alone is sufficient in the majority of cases for the diagnosis of incarceration and strangulation [13]. The same guideline states that ultrasound of the groin or a CT scan can aid decision-making [13]. The role of imaging is not to diagnose the presence of MD, as it has little probable benefit and will not modify the decision to undergo surgery or the surgical attitude.

Most MDs remain asymptomatic [2]. The clinical manifestations of Littre hernia can range from abdominal pain and signs of intestinal obstruction up to enterocutaneous fistula. Similar to enterocutaneous fistula, MD can present with periumbilical cellulitis if it is attached to the abdominal wall [1]. Nevertheless, MD is associated with a much wider array of potential complications, such as hemorrhage, obstruction, diverticulitis, umbilicoenteric fistula, perforation, intussusception, foreign bodies, tumors, peptic ulceration, and Littre hernia [14]. Another type of complication that can occur is inversion of the diverticulum, which may lead to intussusception. Yorishi et al. performed a literature review on cases of inverted Meckel diverticulum and found 31 cases with intussusception out of 74 cases with inverted MD [15].

One important aspect that we wanted to address was treatment. A number of authors have decided to preserve the Meckel diverticulum and not resect it, and one study has published recommendations for when to resect, but these articles fail to follow up with long-term surveillance [2,16,17,18]. Since long-term follow-up is lacking, the outcomes of the preservation of the MD cannot be assessed. It is true that resection of the MD prolongs operative time and may be accompanied by added morbidity, and that it may be the best course of action in selected cases, such as patients in an unstable condition. Our review shows that the presence of hernia complications is high among the presented patients. The presence of heterotopic tissue, ulceration, or tumors in available Littre hernia reports is also very high (42.42%). Additionally, Erdogan reports the presence of neoplasia in the MD on the pathology report [19]. Moreover, an MD with a strangulated or incarcerated hernia may have an insufficient blood supply, which may lead to diverticulitis or the formation of mesodiverticular bands. This, in turn, may lead to new complications such as intestinal obstruction [20]. One important aspect to take into consideration is the reduction of the hernia. Normally, this results in transforming an emergency case into an elective one. In the case of a Littre hernia, reduction of the MD, if it is not incarcerated in the sac, may lead to consecutive complications either in the short or long term.

In the absence of a body of evidence suggesting comparable long-term safety of diverticulum preservation versus MD resection, we cannot issue a recommendation for the preservation of the MD.

Regarding treatment choice, diverticulectomy is usually used in long MD, while wedge resection is typically used in shorter MD with a wider base [21]. Nevertheless, we have found at least one instance in which the terms were improperly used, which is why we decided not to differentiate between the two. We opted for a simpler classification and recorded it as either resection of the MD or bowel resection.

No mortality was recorded in patients undergoing MD resection, while in patients with bowel resection, mortality was 8.7%. Mortality is probably unrelated to the resection technique and should be attributed to acute presentation (mortality in patients with bowel obstruction was 28%). Resection of the MD, or bowel resection, is dictated by the extent of lesions. Nevertheless, patients who require bowel resection may be prone to worse outcomes.

Mesh repair was associated with a shorter postoperative stay and was performed more frequently in patients undergoing MD resection. This may be explained by the use of mesh in less complicated cases and/or without contamination of the site.

We desired to assess the antibiotic regimen in the presence of Littre hernia, but very few authors report whether antibiotic therapy was introduced or maintained.

Few articles report follow-up. Analysis for long-term results is lacking. We can only hypothesize that current rates for recurrence and complications may be similar to the literature data for each hernia type, but considering that most Littre hernias are emergency presentations with frequent strangulation and incarceration and frequent contamination due to bowel resection/MD resection, these rates may be higher.

## 6. Conclusions

In conclusion, Littre hernia is a rare type of hernia containing a Meckel diverticulum. From available cases, it seems that it affects both sexes equally, and literature data may indicate that gender may not be a significant factor in determining prognosis. The most frequent herniation site is femoral, followed by inguinal and umbilical. Most cases are admitted in an emergency setting, and intestinal obstruction is frequently associated with this hernia type. The use of imaging is to aid in the decision for surgery, not to try and diagnose the diverticulum. A minimally invasive approach can be an option even for complicated hernias, especially femoral, inguinal, and umbilical herniations. MD resection or bowel resection is usually employed, depending on the extent of ischemic lesions. Patients undergoing bowel resection may be prone to worse outcomes.

We have found no evidence of long-term safety for preserving the Meckel diverticulum.

## 7. Study Limitation

Limitations of this study may come from the total number of cases assessed and from the interpretation of some of the data whenever it was not concise. Many more cases may have been missed by not searching other databases, as case reports may not always be published in PubMed-indexed journals.

## 8. Future Research Implications

We hope that more reports will be published on these cases. It is unlikely that the site frequency will change significantly in the following years. We encourage extenso reporting of cases with a full description of surgical findings, repair procedure, resection procedure, type of anastomosis if performed, ASA scores, and pathology reports for the surgical specimen. For a more accurate analysis of the outcomes of Littre hernias, we encourage future authors to publish the case report after adequate long-term follow-up and also report on complications, recurrence, and the existence of chronic pain.

## Figures and Tables

**Table 1:** Use of imaging in patients with Littre hernia.

Imaging	* No.	* %
As diagnostic strategy		
X-ray	15	26.32%
X-ray, CT	10	17.54%
X-ray, CT, US	2	3.51%
X-ray, US	5	8.77%
US	8	14.14%
CT	5	8.77%
No imaging	11	19.3%
ND	41	-
Barium meal	1	1.75%
Overall use		
* X-ray		
Yes	32	56.14%
No	25	43.86%
ND	41	-
* CT		
Yes	17	29.82%
No	40	70.18%
ND	41	-
US		
Yes	14	24.56%
No	43	75.44%
ND	41	-

* No—absolute number; %—percentage; X-ray—plain abdominal X-ray; CT—computed tomography; US—ultrasound; ND—no data.

**Table 2:** Hernia complications—described as an absolute number and percentage.

Hernia Complication	* No.	* %
Enterocutaneous fistula	9	9.89%
Incarceration	13	14.29%
Incarcerated bowel with MD	1	1.10%
Incarcerated MD	12	13.19%
Perforation	12	13.19%
Perforated bowel with MD	4	4.40%
Perforated MD	8	8.79%
Strangulation	35	38.46%
Strangulated bowel with MD	6	6.59%
Strangulated MD	29	31.87%
No complication	22	24.18%
Total	91	100.00%

* No—absolute number; %—percentage.

**Table 3:** Overall approach used and by site, expressed as an absolute number and percentage.

Approach	* No.	* %
Overall		
Laparoscopy	5	7.46%
Laparotomy	30	44.78%
* No	32	47.76%
* ND	31	-
Total	67	100%
By site		
Femoral	49	100%
Laparoscopy	2	8.00%
Laparotomy	8	32.00%
* No	15	60.00%
* ND	24	-
Total	25	100%
Incisional	4	100%
Laparotomy	4	100%
Inguinal	27	100%
Laparoscopy	1	4.35%
Laparotomy	7	30.43%
* No	15	65.22%
* ND	4	-
Obturator	3	100%
Laparotomy	3	100%
Spiegel	2	100%
* ND	2	-
Transthoracic	2	100%
Laparotomy	1	50%
* No	1	50%
Umbilical	11	100%
Laparoscopy	2	20.00%
Laparotomy	7	70.00%
* No	1	10.00%
* ND	1	-

* No.—absolute number; %—percentage; ND—no data; No—Incision on herniation site, no laparotomy or laparoscopy.

**Table 4:** Type of resection, technique used, and repair used, expressed as an absolute number and percentage.

Resection	* No.	* %
Bowel resection	23	31.51%
MD resection	46	63.01%
Unresected	4	5.48%
* ND	25	-
Grand Total	73	100.00%
**Technique**		
Stapler	17	40.48%
Manual	24	57.14%
Ileostomy	1	2.38%
* NA	4	9.52%
* ND	52	-
Grand Total	42	100.00%
**Repair**		
Anatomic repair	36	59.02%
Mesh repair	20	32.79%
Hiatus repair	1	1.64%
Yes	2	3.28%
No repair	2	3.28%
* ND	37	-
Grand Total	61	100.00%

* No—absolute number; %—percentage; ND—no data; NA—not applicable; Yes—repair was undertaken, but no data regarding type of repair used.

**Table 5:** The technique used and if anastomosis was carried out in resected cases, expressed as an absolute number and percentage.

Resection/Technique	* No.	* %
Bowel resection	23	23.47
Manual	5	45.45%
* ND	12	-
Stapler	6	54.55%
MD resection	46	46.94
Manual	19	63.33%
* ND	16	-
Stapler	11	36.67%
* ND	25	-
Unresected	4	
Total	98	
**Resection/Anastomosis**		
Yes	18	81.82%
No	4	18.18%
	22	100

* No—absolute number; %—percentage; ND—no data.

**Table 6:** Repair technique with regard to type of resection and outcomes of different resection types, expressed as an absolute number and percentage.

Resection Type/Repair	* No.	* %
Bowel resection	23	
Anatomic repair	14	82.35%
Hiatus repair	1	5.88%
Mesh repair	2	11.76%
* ND	6	
MD Resection	46	
Anatomic repair	22	56.41%
Mesh repair	15	38.46%
* ND	7	
No repair	2	5.13%
* ND	25	
Unresected	4	
Mesh repair	3	75.00%
Yes	1	25.00%
Grand Total	98	
**Resection type/outcomes**		
Bowel resection	23	
Death	2	8.70%
Uneventful	20	86.96%
Wound infection	1	4.35%
Diverticulectomy	46	
* ND	5	
Seroma	1	2.44%
Uneventful	37	90.24%
Wound dehiscence	1	2.44%
Wound infection	2	4.88%
* ND	25	
Unresected	4	
Grand Total	98	

* No—absolute number; %—percentage; ND—no data.

**Table 7:** Postoperative discharge (POD) of different resection types and repair techniques, expressed as average and standard deviation.

Resection	Average POD	Std Dev POD
Bowel resection	13.6	26.24
Diverticulectomy	8.35	7.06
Unresected	3.5	0.5
Grand Total	9.71	15.59
**Repair**		
Anatomic repair	9.79	7.04
Hiatus repair	111	0
Mesh repair	3.73	1.73
ND	6.8	3.82
No repair	10	5
Grand Total	9.71	15.59

**Table 8:** Outcomes of patients with bowel obstruction.

Bowel Obstruction/Outcomes	* No.	* %
* ND	25	-
Without bowel obstruction	47	44
* ND	3	-
Recovered	6	13.64%
Seroma	1	2.27%
Uneventful	36	81.82%
Wound dehiscence	1	2.27%
With bowel obstruction	26	25
Death	7	28%
* ND	1	-
Recovered	1	4%
Uneventful	16	64%
Wound infection	1	4%
Grand Total	98	

* No—absolute number; %—percentage; ND—no data; recovered—the term used in the article, no way of assessing if minor complications existed, which is why it was left unchanged.

**Table 9:** Pathology reports containing metaplasia, dysplasia, and ulceration.

Pathology Report	* No.	* %
Carcinoid tumor	1	3.03%
Gastric mucosa	5	15.15%
Pancreatic tissue	1	3.03%
Heterotopic tissue, not specified	1	3.03%
Neuroendocrine tumor	2	6.06%
Ulceration	4	12.12%
No	19	57.58%
* NA	4	-
* ND	61	-

* No—absolute number; %—percentage; NA—not applicable; ND—no data.

## Appendix A

Search synthax: (((“meckel diverticulum”[MeSH Terms] OR (“meckel”[All Fields] AND “diverticulum”[All Fields]) OR “meckel diverticulum”[All Fields]) AND (“hernia”[MeSH Terms] OR “hernia”[All Fields])) OR (littre[All Fields] AND (“hernia”[MeSH Terms] OR “hernia”[All Fields]))) OR (persistant[All Fields] AND (“vitelline duct”[MeSH Terms] OR (“vitelline”[All Fields] AND “duct”[All Fields]) OR “vitelline duct”[All Fields] OR (“omphalomesenteric”[All Fields] AND “duct”[All Fields]) OR “omphalomesenteric duct”[All Fields]) AND (“hernia”[MeSH Terms] OR “hernia”[All Fields])).

## Appendix B

Full-list of articles included in the analysis can be found in the accompanying spreadsheet and in the references section [2,9,11,14,16,17,18,19,22,23,24,25,26,27,28,29,30,31,32,33,34,35,36,37,38,39,40,41,42,43,44,45,46,47,48,49,50,51,52,53,54,55,56,57,58,59,60,61,62,63,64,65,66,67,68,69,70,71,72,73,74,75,76,77].
